# Genetics and diet shape the relationship between islet function and whole body metabolism

**DOI:** 10.1152/ajpendo.00060.2024

**Published:** 2024-04-03

**Authors:** Belinda Yau, Søren Madsen, Marin E. Nelson, Kristen C. Cooke, Andreas M. Fritzen, Ida H. Thorius, Jacqueline Stöckli, David E. James, Melkam A. Kebede

**Affiliations:** ^1^School of Medical Science, Faculty of Medicine and Health, University of Sydney, Camperdown, New South Wales, Australia; ^2^Charles Perkins Centre, University of Sydney, Camperdown, New South Wales, Australia; ^3^School of Life and Environmental Sciences, University of Sydney, Camperdown, New South Wales, Australia; ^4^Faculty of Medicine and Health, University of Sydney, Camperdown, New South Wales, Australia

**Keywords:** gene-diet interactions, genetic background, high-fat, high-sucrose diet, islet function, keto diet

## Abstract

Despite the fact that genes and the environment are known to play a central role in islet function, our knowledge of how these parameters interact to modulate insulin secretory function remains relatively poor. Presently, we performed ex vivo glucose-stimulated insulin secretion and insulin content assays in islets of 213 mice from 13 inbred mouse strains on chow, Western diet (WD), and a high-fat, carbohydrate-free (KETO) diet. Strikingly, among these 13 strains, islets from the commonly used C57BL/6J mouse strain were the least glucose responsive. Using matched metabolic phenotyping data, we performed correlation analyses of isolated islet parameters and found a positive correlation between basal and glucose-stimulated insulin secretion, but no relationship between insulin secretion and insulin content. Using in vivo metabolic measures, we found that glucose tolerance determines the relationship between ex vivo islet insulin secretion and plasma insulin levels. Finally, we showed that islet glucose-stimulated insulin secretion decreased with KETO in almost all strains, concomitant with broader phenotypic changes, such as increased adiposity and glucose intolerance. This is an important finding as it should caution against the application of KETO diet for beta-cell health. Together these data offer key insights into the intersection of diet and genetic background on islet function and whole body glucose metabolism.

**NEW & NOTEWORTHY** Thirteen strains of mice on chow, Western diet, and high-fat, carbohydrate-free (KETO), correlating whole body phenotypes to ex vivo pancreatic islet functional measurements, were used. The study finds a huge spectrum of functional islet responses and insulin phenotypes across all strains and diets, with the ubiquitous C57Bl/6J mouse exhibiting the lowest secretory response of all strains, highlighting the overall importance of considering genetic background when investigating islet function. Ex vivo basal and stimulated insulin secretion are correlated in the islet, and KETO imparts widescale downregulation of islet insulin secretion.

## Graphical abstract

**Figure:**
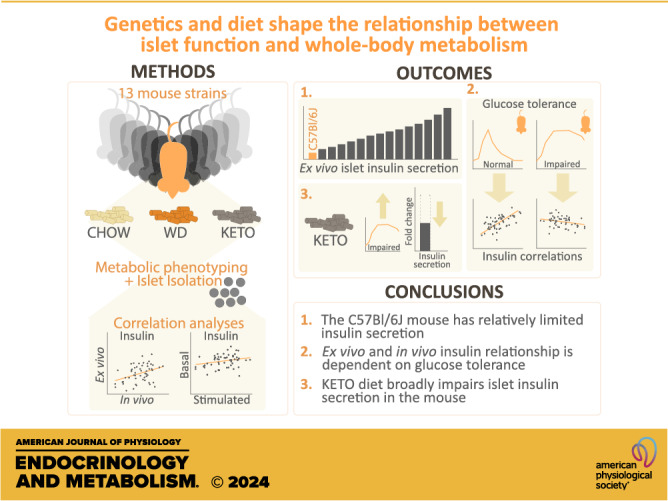

## INTRODUCTION

Insulin secretion from the pancreatic islets occurs in response to a glucose challenge (1). In vivo, glucose-stimulated insulin concentrations can be measured in blood samples at fixed time points after single bolus glucose tolerance testing (oral or intraperitoneal) (2) or continuous infusion during hyperglycaemic clamps (3). Ex vivo, isolated islets are the golden standard model to investigate glucose-stimulated insulin secretion in the experimental animal and are considered an accurate representation of insulin secretion in vivo (4).

Mouse islet studies employing diet interventions often focus on the ubiquitous C57BL/6J mouse strain, which exhibits compensatory beta-cell hyperplasia and hyperinsulinemia in the face of insulin resistance and obesity (5). As such, the C57BL/6J genetic background does not develop beta-cell failure to overt diabetes. There is therefore a severe underappreciation of islet function in a broader range of genetic backgrounds, particularly in non-C57BL/6 strains with no specific indication of diabetes (and beta-cell dysfunction). Recently, metabolic phenotypic characterization of seven inbred strains fed chow (CHOW) or high-fat/sucrose Western diet (WD) alongside ex vivo islet parameters was correlated with deep islet proteomics to identify key protein pathways implicated in metabolic dysfunction (6). Similarly, correlative analysis of islet proteomics and metabolic phenotyping in eight founder strains of the Collaborative Cross on CHOW and WD resulted in the identification of the dopamine synthesis pathway as a negative regulator of insulin secretion (7). More recently, using the same eight founder strains of the Collaborative Cross, the remarkable diversity of nutrient-evoked islet Ca^2+^ responses was uncovered (8).

Variations of the ketogenic diet have recently garnered significant attention due to its potential roles in weight loss (9), glucose resensitization (10), and increased life span in mice (11). Significantly, the effects of the ketogenic diet as a dietary intervention in people with obesity and/or diabetes have led to its primary assertion as a model of beta-cell rest, in which the consequence is an overall reduction in glycaemic excursions, resulting in reduced hyperinsulinemia and increased beta-cell preservation (12). However, diet studies investigating islet function in mice are mostly performed in CHOW and high-fat, high-sugar variations of the WD (13), with relatively few studies assessing the role of ketogenic diet variants, particularly in the nonobese, nondiabetic context.

The present study employs a panel of 13 genetically diverse inbred mouse strains, selected for their wide spectrum of metabolic and insulin secretory responses. Previous assessments of these mice, in CHOW and WD comparisons, focused primarily on strain-by-diet interactions in the context of glucose utilization in insulin-resistant states and uncovered distinct metabolic characteristics in adipose and skeletal muscle tissue (14). Here we employ an islet-centric analysis of the same mice with the addition of a high-fat, carbohydrate-free diet (KETO) detailing measures of islet function from isolated islets alongside previously established whole body glucose and insulin-related parameters. Strain comparisons of islet function indicate a large spectrum of ex vivo insulin secretion capacities incongruous with conventional C57BL/6J responses, where most strains display higher glucose responsiveness on insulin secretion compared to C57BL/6J islets. Correlation of isolated islet parameters finds an intrinsic relationship between basal and glucose-stimulated insulin secretion, while analyses between islet parameters and whole body phenotypes identified significant correlations between ex vivo insulin secretion and in vivo insulin outcomes in glucose-tolerant animals only. Significantly, a comparison of ex vivo parameters on KETO and CHOW diets also revealed an overall deleterious effect of KETO on insulin secretory capacity.

## METHODS

### Animals

Islets were isolated from male animals previously published (14) with the addition of animals fed a high-fat, carbohydrate-free diet. In short, mice were housed at 23°C on a 12-h light-dark cycle with free access to food and water. The following strains were acquired from Australian BioResources (Moss Vale, NSW, Australia): 129X1/SvJ, C57Bl/6J, ILSXISS50, ILSXISS89, ILSXISS97, ILSXISS98, BXD34/TyJ, and BXH9/TyJ. The following animals were from the Animal Resources Center (Perth, WA, Australia): A/J, Balb/C, DBA, NOD/ShiltJ, and AKR and were bred in-house at the University of Sydney. Experiments were performed in accordance with National Health and Medical Research Council (Australia) guidelines and under the approval of The University of Sydney Animal Ethics Committee. A range of 3–12 animals of each strain was placed on separate diets; from age 8 to 10 wk of age, mice were fed either a standard laboratory chow containing 12% calories from fat, 65% calories from carbohydrate, 23% calories from protein (Irradiated Rat and Mouse Diet; Specialty Feeds, Glen Forest, WA, Australia); a high-fat, high-sucrose Western diet (WD) made in-house containing 45% calories from fat, 36% calories from carbohydrate and 19% calories from protein; or a carbohydrate-free, high-fat diet (KETO) made in-house containing 91% calories from fat and 9% from protein (not containing any carbohydrate) for 8 wk. Specifically, the WD contained 3.5% g cellulose, 4.5% g bran, 13% g cornstarch, 21% g sucrose, 16.5% g casein, 3.4% g gelatine, 2.6% g safflower oil, 18.6% g lard, 1.2% g AIN-93 vitamin mix (MP Biomedicals), 4.95% g AIN-93 mineral mix (MP Biomedicals), 0.36% g choline, and 0.3% g l-cysteine. The carbohydrate-free, high-fat diet contained: 13% g casein, 3.4% g gelatine, 2.2% g safflower oil, 70% g lard, 2.0% g AIN-93 vitamin mix (MP Biomedicals), and 8.4% g AIN-93 mineral mix (MP Biomedicals).

### Metabolic Phenotyping

Metabolic phenotyping was previously published for the CHOW and WD mice (14). In short, blood was collected from the saphenous vein after 2 h fast into EDTA-coated tubes and centrifuged at 2,000 *g* for 15 min. Body composition was determined by EchoMRI-900 (EchoMRI Corporation Pte Ltd., Singapore) after 7 wk of diet. The same week, oral glucose tolerance tests were performed after a 6-h fast at 2 mg glucose/kg bodyweight. Blood glucose was measured from the tail vein with a glucose monitor (Accu-Chek, Roche Diabetes Care, NSW, Australia) at 0, 15, 30, 45, 60, 90, and 120 min after a glucose gavage. Blood insulin levels were measured in whole blood at 0, 5, 10, and 30 min with Insulin Mouse Ultra Sensitive ELISA (Crystal Chem, Elk Grove Village, IL). The assay was then performed according to the manufacturer’s protocol with the exception of using whole blood.

An intraperitoneal insulin tolerance test was performed on mice after 6 wk of diet after a 2-h fast via intraperitoneal injection of insulin in PBS at 1 U/kg lean mass. Blood glucose was measured as above at 0, 5, 10, 20, and 30 min after insulin injection.

### Pancreatic Islet Isolation

Twenty to thirty islets per mouse were harvested following a modified islet isolation protocol: the excised pancreata of euthanized mice were externally injected with a 2 mL solution of Liberase and collagenase (Roche). The partially inflated pancreas was then dissected into 1-mm pieces using dissection scissors and incubated at 37°C in an additional 1 mL of the liberase/collagenase solution for 12 min. The digested pancreas tissue was then processed as previously described (6). Briefly, the pancreas was washed in islet wash buffer (HBSS containing 10 mM HEPES, and 0.1% BSA), passed through a mesh filter, and then layered onto a Histopaque gradient to collect islets at the Histopaque/HBSS interface. Islets were recovered in Islet Media (RPMI 1640, 10% FBS, and 1% penicillin/streptomycin) for 1 h before an ex vivo glucose-stimulated insulin secretion assay.

### Glucose-Stimulated Insulin Secretion Assay

Islets were transferred to 2.8 mM glucose Krebs-Ringer buffer supplemented with 10 mM HEPES (KRBH) in 3.5-cm untreated petri dishes for 1 h (prebasal condition). Islets were then separated and collected into 1.5-mL tubes with a total volume of 150 µL of KRBH containing 2.8 mM glucose (basal secretion) or 16.7 mM glucose (stimulated secretion) for 1 h. The supernatant was collected, and islets were snap frozen in 50 µL islet lysis buffer (100 mM Tris, 300 mM NaCl, 10 mM NaF, and 2 mM sodium orthovanadate) for total islet insulin content analysis.

### Insulin Secretion and Content Measurements

The commercial HTRF Ultra-Sensitive Insulin Assay Kit (Cisbio) was used to measure insulin secretion and insulin content from islet samples as per manufacturer’s instructions. Islet insulin secretion has been expressed as nanograms of insulin secreted per islet, alongside the fold change of stimulated secretion over basal secretion. Islet insulin content has been expressed as nanograms of insulin per islet.

### Correlation and Statistical Analysis

Correlation analyses and statistical comparisons (one-way ANOVA with Sidak’s multiple comparison test, two-way ANOVA with Tukey’s multiple comparison test) were performed using GraphPad Prism 8 (GraphPad Software).

## RESULTS

### The Conventional C57BL/6J Mouse Model Offers Limited Insight into Islet Insulin Measures

**Figure 1.**
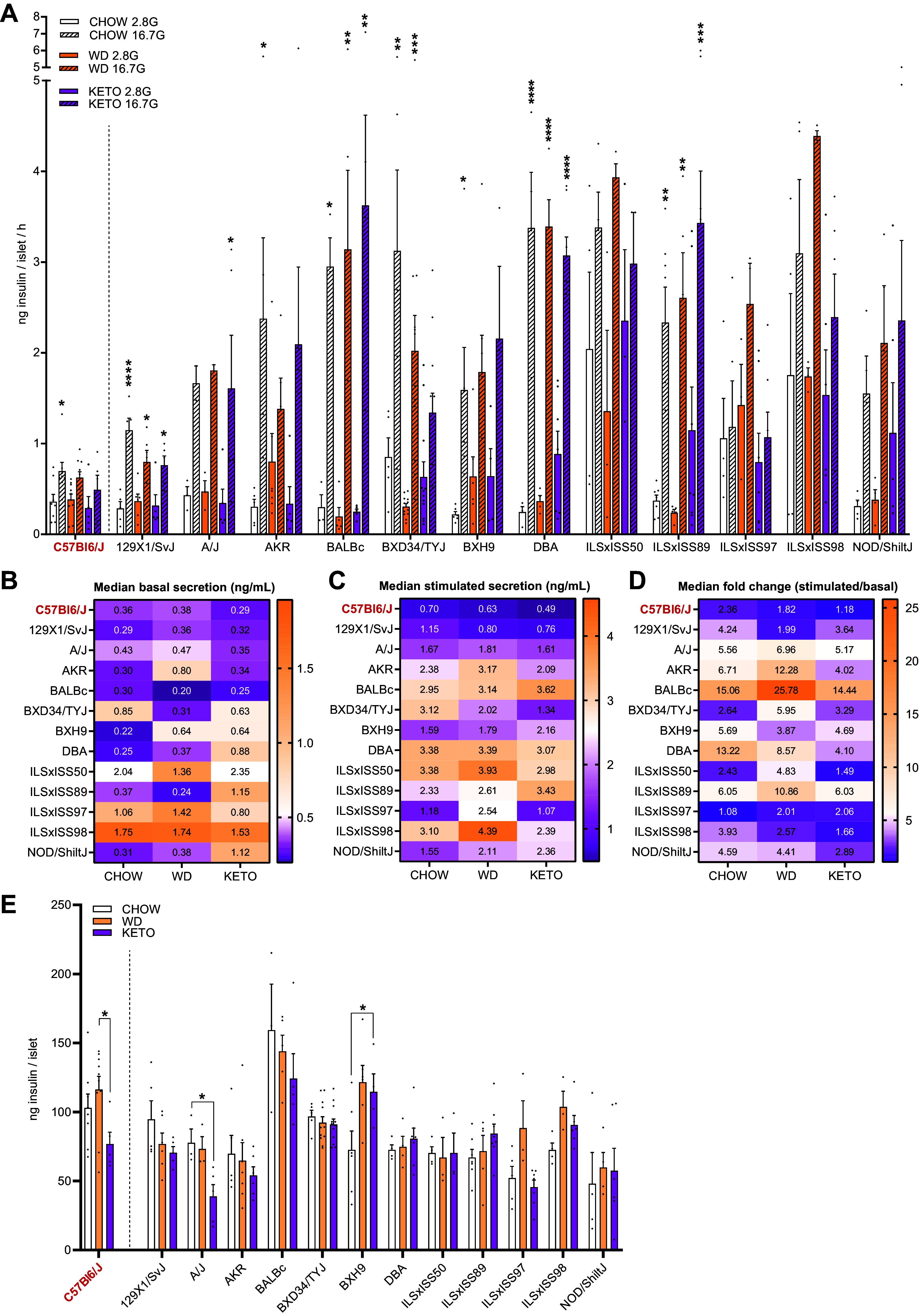
Variable islet secretion and content measures across strains and diets. *A*: insulin secreted by islets isolated from CHOW, Western diet (WD), and high-fat, carbohydrate-free (KETO)-fed mice of all strains during ex vivo glucose stimulation at 2.8 mM and 16.7 mM glucose concentrations. Ten islets of 1–3 replicates per condition were averaged, and insulin secretion are expressed as ng insulin per islet per 1 h of secretion assay. Error bars represent mean and standard deviation. *B* and *C*: median basal secretion at 2.8 mM glucose (*B*) and high-glucose-stimulated secretion at 16.7 mM glucose (*C*) are reported as ng insulin secreted per mL in 1 h of culture. *D*: median fold change of stimulated secretion at 16.7 mM glucose/basal secretion at 2.8 mM glucose. *E*: total insulin content of islets isolated from CHOW, WD, and KETO-fed mice of all strains after glucose-stimulated insulin secretion assays. Ten islets of 1–3 replicates were averaged per mouse, and insulin concentrations are expressed as ng insulin per islet. Asterisk denotes a significant increase in insulin secretion compared to basal, where *P < 0.05, **P < 0.01, ***P < 0.001, ****P < 0.0001.

The C57BL/6J mouse model is probably the most extensively utilized model for studying pancreatic islet function and insulin secretion. Recent studies utilizing mouse strains to investigate aspects of glucose-stimulated insulin secretion still rely heavily on the C57BL/6J mouse model (15, 16). We have previously utilized a panel of inbred mouse strains to interrogate adipose and muscle-specific insulin action and the whole body consequences of this (14). This is a powerful approach as it establishes a spectrum of traits and allows for the identification of associations otherwise impossible to find between (two) discrete groups. In this study, we have expanded on this previous work to include animals fed KETO and carefully characterized islet function ex vivo across 13 inbred mouse strains. Importantly, basal, and glucose-stimulated insulin secretion observed from C57BL/6J islets in our present study was comparable to that observed in previous studies (17, 18). In the CHOW condition, high-glucose-stimulated insulin secretion was significantly higher in 8 out of 13 strains compared to C57BL/6J mice (Fig. 1, *A*–*C*). In fact, C57BL/6J islets showed nominally the lowest glucose-stimulated insulin secretion per islet independent of the diet of all strains. To highlight the relatively small insulin responses observed in C57BL/6J mice, chow-fed C57BL/6J mice exhibited a 2.4-fold change in stimulated/basal insulin secretion, whereas BALB/C and DBA had ∼15- and ∼13-fold change in glucose responsiveness on insulin secretion on CHOW diet, respectively (Fig. 1*D*). Notably, of the 13 strains investigated in this study, 11 reported higher stimulated/basal insulin secretion fold changes on CHOW diet compared to the C57BL/6J strain, and all 13 strains reported higher fold change secretion on WD or KETO (Fig. 1*D*).

To determine if this strain variation in insulin secretion per islet could be accounted for by differences in total insulin, we next measured insulin content. Islet insulin content in C57BL/6J mice on CHOW was again comparable to that previously described (19), and insulin content varied greatly between mouse strains and less within a strain between diets (Fig. 1*E*). For instance, there was a 3-fold difference between chow-fed BALB/C and NOD/ShiltJ on the same diet (∼160 vs. ∼50 ng/islet), which represented the highest and lowest islet insulin content observed (Fig. 1*E*) in CHOW-fed mice. However, 10 of 13 strains did not have significantly different islet insulin content compared to C57BL/6J on the chow diet.

After 8 wk of WD or KETO, no differences in islet insulin secretion were observed in C57BL/6J mice compared to CHOW; however, total islet insulin content was increased and decreased, respectively. These opposing effects in the C57BL/6J pancreatic islet resulted in a significantly altered total islet insulin content between WD and KETO (116.3 ± 29.9 ng/islet WD to 76.83 ± 18.98 ng/islet KETO; *P* < 0.05). Notably, this divergent diet response was not observed in any other investigated strain. Across all strains, WD only significantly increased islet insulin content in BXH9 (72.53 ± 33.62 ng/islet CHOW to 121.6 ± 29.65 ng/islet WD; *P* < 0.05), while KETO significantly decreased islet insulin content in A/J (77.59 ± 17.50 ng/islet CHOW to 35.08 ± 20.11 ng/islet KETO; *P* < 0.05), with overall islet insulin content remaining stable within strains regardless of diet intervention (Fig. 1*E*). These data show that the C57BL/6J islets are not representative of mouse islets in general and that the responses to different diets are dictated by the underlying genetic background of each strain. Intriguingly, C57BL/6J islets were the least glucose-responsive islets in this study.

### Islet Glucose-Stimulated Insulin Secretion Is Correlated with Basal Secretion Independent of Total Insulin Content

**Figure 2.**
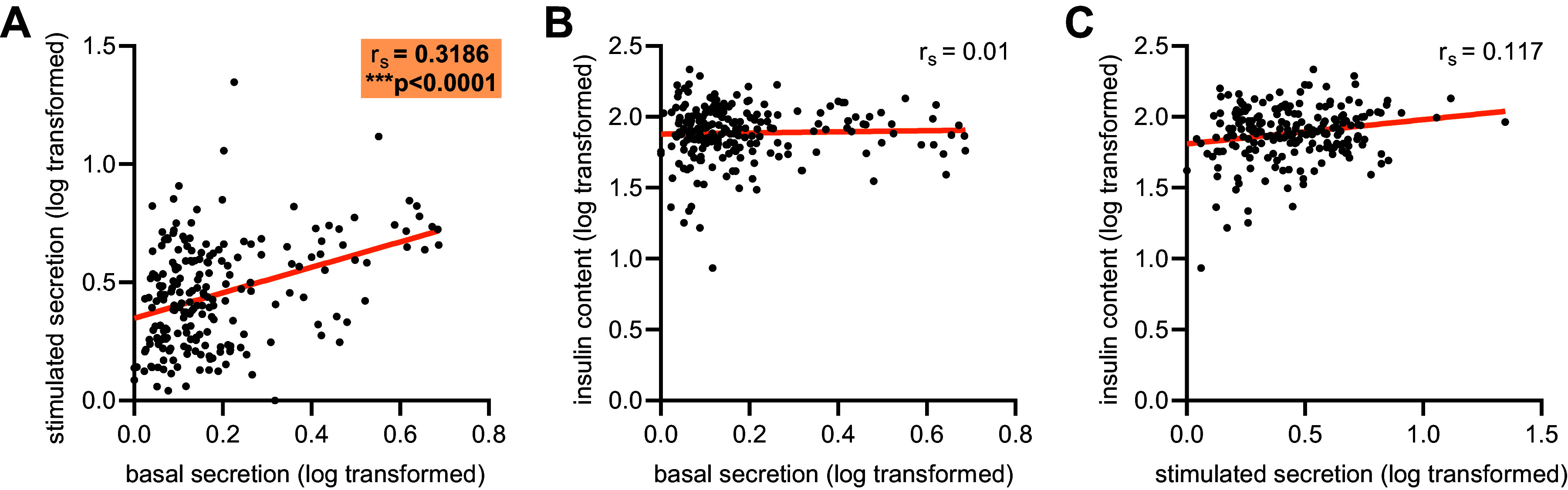
Correlations between insulin secretion and insulin content in isolated islets. *A*: Spearman’s rank correlation (*r*_s_) of log-transformed basal insulin secretion and stimulated insulin secretion in ex vivo islets. ***P < 0.0001. *B*: Spearman’s rank correlation of log-transformed basal insulin secretion and total insulin content in ex vivo islets. *C*: Spearman’s rank correlation of log-transformed stimulated insulin secretion and total insulin content in ex vivo islets. Each dot represents an individual mouse.

There was a significant positive correlation between basal and glucose-stimulated insulin secretion measured in islets from the same mouse independent of strain or diet (Fig. 2*A*, Spearman’s rank coefficient = 0.3125, *P* < 0.0001). In contrast, neither basal insulin secretion nor glucose-stimulated insulin secretion correlated with total islet insulin content (Fig. 2, *B* and *C*, Spearman’s rank coefficients = −0.002 and −0.0106, respectively) across all mice, irrespective of diet.

### The Relationship between In Vivo Insulin and Ex Vivo Insulin Secretion in Isolated Islets Is Lost with Impaired Glucose Tolerance

**Figure 3.**
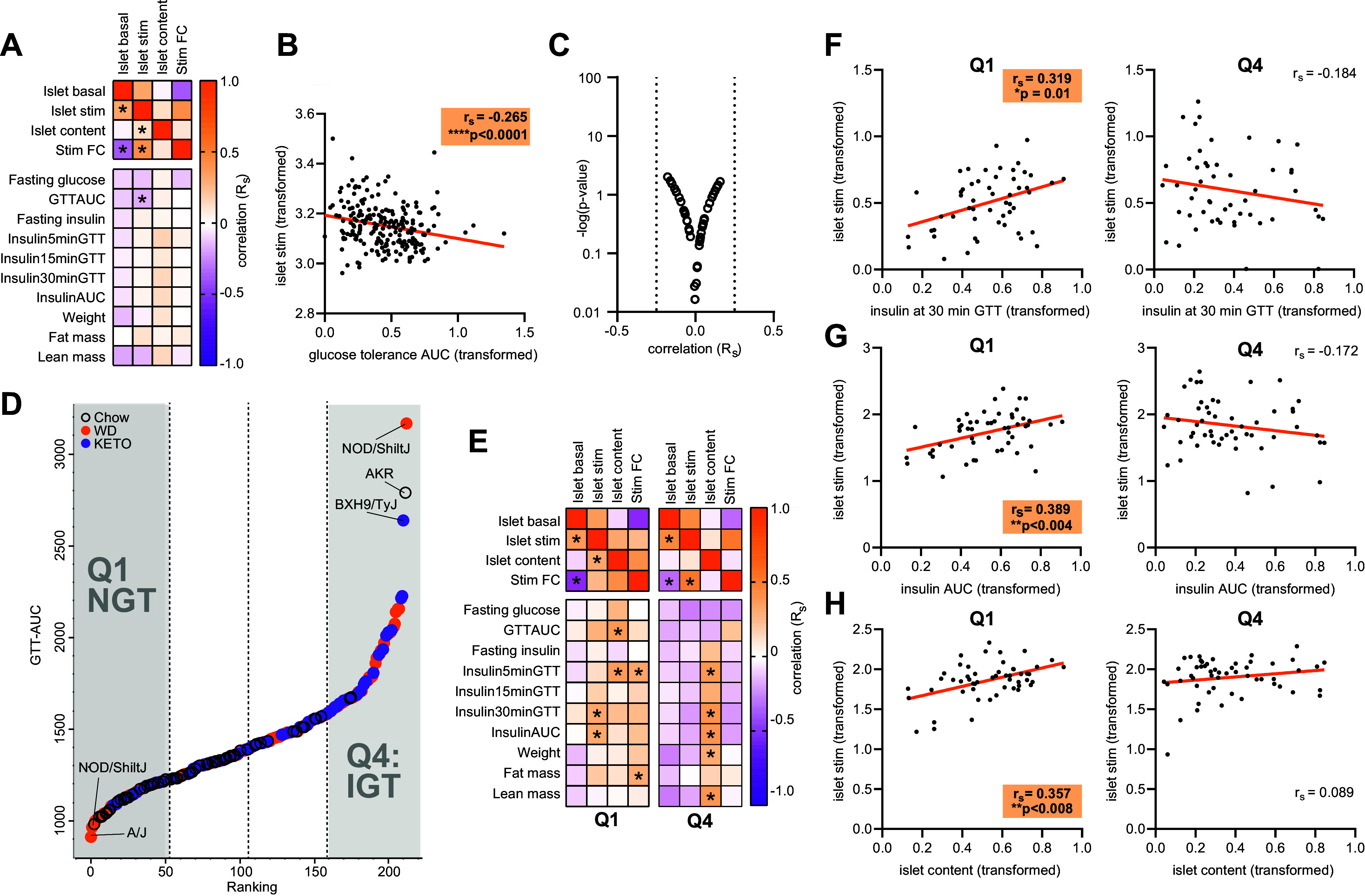
Correlations between ex vivo islet measures and in vivo phenotypic measures across strains and diets. *A*: heatmap of Spearman’s rank (*r*_s_) correlation coefficients between islet-centric measures and whole body phenotypic parameters in all mice. *P < 0.05, significance. *B*: Spearman’s rank correlation of log-transformed whole body in vivo glucose tolerance area under the curve (AUC) and stimulated insulin secretion in ex vivo islets. ****P < 0.0001. *C*: volcano plot of correlated traits in all mice, dotted line denotes Spearman’s rank correlation coefficient cutoff of ± 0.3. *D*: all mice ranked by in vivo glucose tolerance test AUC, with quartiles 1 (Q1) and 4 (Q4) highlighted for further analysis. *E*: heatmap of Spearman’s rank correlation coefficients of islet-centric measures and whole body phenotypic parameters in mice only subscribed to either Q1 or Q4. *P < 0.05, significance. *F*: Spearman’s rank correlation of log-transformed stimulated insulin secretion in ex vivo islets and in vivo plasma insulin at 30 min during glucose tolerance test in mice of either Q1 or Q4. *P < 0.05. *G*: Spearman’s rank correlation of log-transformed stimulated insulin secretion in ex vivo islets and in vivo plasma insulin AUC during glucose tolerance test in mice of either Q1 or Q4. **P < 0.01. *H*: Spearman’s rank correlation of log-transformed stimulated insulin secretion and log-transformed insulin content in ex vivo islets of either Q1 or Q4. **P < 0.01. WD, Western; KETO, high-fat, carbohydrate; GTT, glucose tolerance test; NGT, normal glucose tolerance; IGT, impaired glucose tolerance; FC, fold change.

To identify potential relationships between ex vivo islet parameters and whole body metabolism, whole body phenotypes, such as fasting insulin, parameters during an oral glucose tolerance test (GTT), and body composition, were correlated against ex vivo islet measurements in all mice. Overall, glucose tolerance, measured as the area under the curve of blood glucose during GTT (AUC), was the only whole body measure that correlated significantly with ex vivo islet stimulated secretion across the entire data set (Fig. 3, *A* and *B*; Spearman’s rank coefficient = −0.265, *P* < 0.0001). However, no correlations reached a Spearman’s rank coefficient cutoff of ±0.30 (Fig. 3*C*).

Pancreatic beta-cells can undergo significant regulation of insulin secretion in response to changes in peripheral insulin resistance, a phenomenon termed beta-cell compensation. To understand how ex vivo islet measurements might reflect changes in in vivo metabolic status, mice were ranked by glucose tolerance AUC to stratify them into quartiles. Q1 contained the most glucose-tolerant mice (normal glucose tolerance), and Q4 contained mice with the most “impaired glucose tolerance” (Fig. 3*D*). Separately within Q1 and Q4, in vivo parameters were correlated with ex vivo islet measurements to explore relationships within extreme metabolic states. Overall, islet parameters shifted from correlating positively with whole body parameters in the healthy Q1 to negative in the metabolic impaired state, Q4 (Fig. 3*E*). Interestingly, the type of islet parameter also shifted between metabolic state, so that the total insulin content association with an impaired metabolic state was less pronounced in the healthy state. To highlight these differences, there was a significant positive correlation between islet glucose-stimulated secretion and blood insulin during the GTT at 30 min (Spearman’s rank coefficient = 0.319, *P* = 0.01, Fig. 3*F*) in Q1, as well as islet glucose-stimulated secretion and insulin AUC during GTT (Spearman’s rank coefficient = 0.389, *P* < 0.005, Fig. 3*G*). In contrast, there were no correlations between these measures in Q4 (Fig. 3, *F* and *G*). Ex vivo islet insulin content significantly correlated with multiple measures of in vivo blood insulin during the GTT, as well as total weight and lean mass (Fig. 3*E*) in the impaired Q4. Interestingly, islet glucose-stimulated secretion significantly correlated with total islet insulin content in Q1 only (Spearman’s rank coefficient = 0.357, *P* = 0.008, Fig. 3*H*), which contrasted the overall pattern across the entire data set (Fig. 2*C*). Together these data show that islet functions associate with different metabolic states.

### KETO Impairs Pancreatic Beta-Cell Function

While islet function after WD and its various forms have previously been investigated across multiple genetic backgrounds (6, 20, 21), the effects of KETO on pancreatic islet function have not been well described. Furthermore, there is also a lack of metabolic phenotyping data in the comparison of KETO and CHOW in the context of both impaired metabolic states but also in a nonobese, nondiabetic status, regardless of genetic background as most prior studies introduced KETO in obese, metabolically compromised mice fed WD (9, 22).

**Figure 4.**
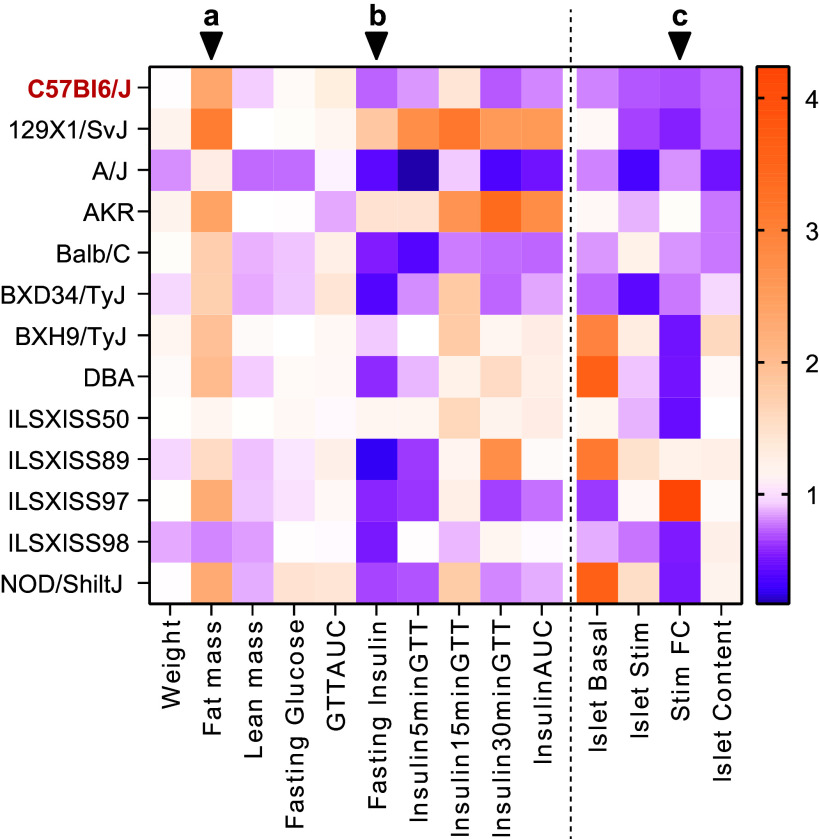
Changes to whole body and islet phenotypes in high-fat, carbohydrate-free (KETO) compared to CHOW across strains. Values represent fold changes of averaged phenotypic measures in KETO over CHOW mice of each strain. AUC, area under the curve; GTT, glucose tolerance test; FC, fold change. ^a,b,c^Variations in fat mass, fasting plasma insulin, and islet secretion fold change, respectively.

To address pancreatic beta-cell function after KETO, we focused on in vivo phenotyping parameters and ex vivo islet measurements and expressed them as the difference between KETO and CHOW diets. As previously reported (9, 23), KETO increased fat mass in C57BL/6J mice (Fig. 4), and this was a general trend, as 11 out of 13 strains displayed increased fat mass relative to chow-fed mice. Importantly, glucose-stimulated insulin secretion (fold over basal) was decreased in islets from animals fed KETO in 10 out of 13 strains relative to CHOW (Fig. 4), concomitant with reduced fasting insulin (Fig. 4). These data show that KETO significantly impairs relative stimulated insulin secretion in islets independently of genetic background. Thus, similar to that described in other peripheral tissues, this implies an adaptive response that generates an islet-specific metabolic inflexibility.

## DISCUSSION

Islet function plays a key role in maintaining metabolic homeostasis and dysfunctional islets are a hallmark of type 2 diabetes. In rodent models, functional assessment of islets after metabolic stressors has primarily been done in the C57BL/6J mouse strain. This study sought to characterize islet function in a panel of inbred mouse strains exposed to three different diets. This revealed a wide spectrum of responses to diet that would not be possible to assess in a single mouse strain. Across the 13 genetically diverse strains, a range of 2-fold to 25-fold glucose-stimulated insulin secretion was observed, with the C57BL/6J strain surprisingly exhibiting the lowest stimulated secretion of all strains. Ex vivo islet insulin secretion in response to both WD and KETO diets varied greatly, emphasizing the heterogenous effects of diet on different genetic backgrounds.

Although islet replicates were small across our sample size, this is an important finding for islet researchers moving forward, as assumptions of islet biology may be distorted by single mouse strain studies. Additionally, where variations of WD and KETO have been previously described to increase and decrease insulin content in C57BL/6J mice, respectively (24–26), a phenomenon also observed in the present study, it is now apparent that this is not representative of a broader genetic population. Furthermore, the C57BL/6J mouse has mutations in the *Nnt* gene that may affect glucose-stimulated insulin secretion (27–29). As such, care should be taken when considering the human relevance to these C57BL/6J responses to KETO, where diet-induced changes to insulin content are more likely primarily driven by genetic background. Finally, our experimental setup did not allow for accessing parameters such as islet size, number, or density, and these considerations in future work will offer additional insight into strain-dependent variation in the islet.

### Relationship between Basal and Glucose Stimulated Insulin Secretion in Isolated Islets

Our approach revealed a significant correlation between basal and glucose-stimulated insulin secretion independent of strain or diet, and this suggests there may exist intrinsic modulation of secretion capacity within a single individual. That is, in individuals where basal insulin secretion is relatively low, it might be predicted that glucose-stimulated insulin secretion may also be relatively low and vice versa. Whether this is a biological limitation at the beta-cell level, potentially driven by fixed calcium channel (30) or levels of regulators of exocytosis (31), or a regulated phenomenon, is yet unknown and an important area for future studies.

The relationship we observe between islet insulin secretion and insulin content dependent on metabolic status is interesting. In humans, positive correlations between islet insulin content and both first- and second-phase secretion has been reported (32). In our data, the reduced correlation between insulin secretion and insulin content specifically in animals with impaired glucose tolerance may be indicative of beta-cell dysfunction at the level of the insulin secretory pathway uncoupling these variables. While there are many transgenic mouse models of beta-cell insulin content deficiency that result in reduced insulin secretion (17, 33), there are also reported models of severe insulin content depletion with no impairment in single-stimulus insulin secretion (34). Additionally, we found in the present study that insulin content was generally maintained within a strain when subjected to dietary intervention, despite significant changes in secretory output, also supports independent regulation of these parameters.

### The Relationship between Plasma Insulin In Vivo and Ex Vivo Insulin Secretion Is Dependent on Glucose Tolerance

Plasma insulin measurements observed during the GTT reflect beta-cell insulin secretion alongside hepatic and extrahepatic insulin clearance (35). Plasma insulin remains a key indicator of insulin-dependent glucose disposal (36) but is arguably not a good measure of beta-cell function (37), at least not without taking individual metabolic status into account. More importantly, our ex vivo glucose-stimulated insulin secretion assays in islets do not consider neurotransmitter or incretin effects during the GTT (38, 39). Nonetheless, there was a positive correlation between plasma insulin and islet-stimulated insulin secretion in the mice with normal glucose tolerance in our data set suggesting there are specific conditions in which plasma insulin levels could reflect beta-cell function. As there also exists a significantly positive correlation between blood glucose AUC during a GTT and an insulin tolerance test (a measure of insulin sensitivity) measured within the same mice (14), loss of this in vivo*/*ex vivo insulin correlation in impaired glucose tolerance mice strongly suggests that peripheral insulin resistance disrupts this relationship. That is, in individuals where peripheral tissue insulin sensitivity is high, beta-cell insulin secretion may be more accurately represented by plasma insulin concentrations. Concurrently, in the insulin-resistant state, this may be indicative of hyperinsulinemia or reflecting larger variabilities in plasma insulin, where the range of insulin secretion from islets required to maintain euglycemia is now much larger. The same can be said of the human islet, with evidence that ex vivo islet insulin secretion readily corresponds to in vivo secretion, except in type 2 diabetic individuals (19), with the caveat that these comparisons refer to perifused islets and intravenous glucose infusions.

### Effect of KETO on Pancreatic Beta-Cell Function

In humans, applications of low-carbohydrate diets in the treatment of type 2 diabetes offer favorable outcomes; reductions in body mass are associated with improvement in HbA_1c_ levels, lower fasting blood glucose, and reduced reliance on glucose-lowering medication (40–42). In the nonobese, nondiabetic setting, where the former positive outcomes are not required; however, KETO remains an unsubstantiated intervention. A study of a low-carbohydrate diet in normal-weight, healthy human individuals elevated homeostatic model assessment-insulin resistance, homeostatic model assessment-β, and plasma C-peptide levels, indicating dysregulated glucose homeostasis (43).

In mice, ketogenic diets exert similar outcomes; obese animals placed on a low-carbohydrate, high-fat diet see reductions in body mass and improved glucose tolerance (9, 22). In normal-weight mice, however, a ketogenic diet can induce increased dyslipidemia (44), inflammation (45), hepatic insulin resistance (23), and glucose intolerance (45). In our present mouse study, impaired glucose tolerance was a ubiquitous finding in KETO with most strains exhibiting worsening of glucose tolerance when compared to CHOW. This is perhaps unsurprising considering the administration of glucose in a system that has metabolically adapted to encountering little to no carbohydrates. This is readily reflected in the reductions of fasting plasma insulin in KETO compared to CHOW in almost all strains of our present study. In C57BL/6J mice on KETO, but also on other forms of low-carbohydrate diets, impaired glucose tolerance is primarily due to hepatic insulin resistance, and rarely impaired glucose clearance or peripheral glucose uptake (23, 46, 47).

At the level of the islet, KETO downregulates both beta-cell and alpha-cell mass, and thus total islet size, in the C57BL/6J mouse (47, 48). Furthermore, feeding a low-carbohydrate diet equivalent to KETO in NZO mice, which become obese and diabetic on CHOW, gave no beneficial effects to beta-cell function or changes to beta-cell mass (49). In the mouse strains in our present analysis, insulin content did not change consistently in either direction in response to KETO, suggesting this response is strain dependent. In contrast, insulin secretion was reduced in almost all strains when compared to CHOW, suggesting KETO induces an overall downregulation of the beta-cell secretory response independent of genetics and a loss of metabolic flexibility in the islet. It is well described that while acute fatty acid stimulation of beta-cells results in increased insulin secretion (50), chronic fatty acid exposure (lipotoxicity) in beta-cells inhibits both glucose-stimulated insulin secretion and insulin biosynthesis, downregulating not only insulin and PDX-1 gene expression (51) but also genes involved in lipid and glucose metabolism (52). In the hyperinsulinaemic contexts of obesity-driven insulin resistance, reduced beta-cell secretory function could be reasonably beneficial when paired with the reduced insulin demand of the KETO environment; however, reinstatement of a carbohydrate source would undoubtedly expose an insulin secretory defect. This is particularly significant when considering KETO adherence in human interventions is almost certainly imperfect, and individuals subject to even small amounts of carbohydrates after KETO would likely exhibit impaired insulin secretory function.

### Conclusions

Together these data demonstrate the relatively limited capacity of ex vivo C57BL/6J islet secretion when compared to a broader genetic population. The results highlight not only the profound variation of insulin secretion and insulin content across different mouse genetic backgrounds but also demonstrate the large spectrum of the islet physiological response to environmental perturbation. Significantly, the data report correlations between ex vivo islet insulin secretion and in vivo plasma insulin concentrations and highlight how metabolic dysfunction may destabilize this relationship. Finally, the findings note a broad secretory impairment in mouse beta-cells subject to a high-fat, carbohydrate-free ketogenic diet, while underscoring the limitations that face islet metabolic studies that rely on standalone genetic mouse models.

## DATA AVAILABILITY

Data will be made available upon reasonable request.

## GRANTS

This work was supported by National Health and Medical Research Council Grant GNT1086851 and Australian Research Council (ARC) Grant FL200100096 (to D.E.J.). D. E. J. is supported by an ARC Laureate Fellowship.

## DISCLOSURES

No conflicts of interest, financial or otherwise, are declared by the authors.

## AUTHOR CONTRIBUTIONS

B.Y., S.M., M.E.H., A.M.F., I.H.T., J.S., D.E.J., and M.A.K. conceived and designed research; B.Y., S.M., M.E.H., K.C.C., A.M.F., I.H.T., J.S., and D.E.J. performed experiments; B.Y., S.M., and M.E.H. analyzed data; B.Y., S.M., D.E.J., and M.A.K. interpreted results of experiments; B.Y. prepared figures; B.Y. drafted manuscript; B.Y., S.M., M.E.H., A.M.F., I.H.T., J.S., D.E.J., and M.A.K. edited and revised manuscript; B.Y., S.M., M.E.H., K.C.C., A.M.F., I.H.T., J.S., D.E.J., and M.A.K. approved final version of manuscript.
